# The moral code in Islam and organ donation in Western countries: reinterpreting religious scriptures to meet utilitarian medical objectives

**DOI:** 10.1186/1747-5341-9-11

**Published:** 2014-06-02

**Authors:** Mohamed Y Rady, Joseph L Verheijde

**Affiliations:** 1 Department of Critical Care Medicine, Mayo Clinic Hospital, Mayo Clinic, Phoenix, Arizona, USA; 2 Department of Physical Medicine and Rehabilitation, Mayo Clinic, Phoenix, Arizona, USA

**Keywords:** Death, Organ donation, Organ procurement, Transplantation, Islamic moral code, Religion, Culture

## Abstract

End-of-life organ donation is controversial in Islam. The controversy stems from: (1) scientifically flawed medical criteria of death determination; (2) invasive perimortem procedures for preserving transplantable organs; and (3) incomplete disclosure of information to consenting donors and families. Data from a survey of Muslims residing in Western countries have shown that the interpretation of religious scriptures and advice of faith leaders were major barriers to willingness for organ donation. Transplant advocates have proposed corrective interventions: (1) reinterpreting religious scriptures, (2) reeducating faith leaders, and (3) utilizing media campaigns to overcome religious barriers in Muslim communities. This proposal disregards the intensifying scientific, legal, and ethical controversies in Western societies about the medical criteria of death determination in donors. It would also violate the dignity and inviolability of human life which are pertinent values incorporated in the Islamic moral code. Reinterpreting religious scriptures to serve the utilitarian objectives of a controversial end-of-life practice, perceived to be socially desirable, transgresses the Islamic moral code. It may also have deleterious practical consequences, as donors can suffer harm before death. The negative normative consequences of utilitarian secular moral reasoning reset the Islamic moral code upholding the sanctity and dignity of human life.

## Introduction

Scientific and scholarly debates about defining death for organ procurement purposes have intensified [1]. The current legal definition requires the irreversible cessation of all functions of the entire brain or the irreversible cessation of circulatory and respiratory functions [1]. All Abrahamic faith traditions (Judaism, Christianity, and Islam) have expressed support for this definition of death, assuming it is supported by scientific evidence [2]. If truly death has occurred, then current timing of organ procurement is appropriate and permissible (ie, organ procurement “*ex cadavere*”) [3]. However, the medical literature is unsettled about the brain and circulatory criteria of death determination [1,4-8]. Permanent unconsciousness and cessation of brainstem reflexes (including apnea) constitute the brain criterion of death, while the circulatory criterion is determined by 2 to 5 minutes of absent arterial pulse [1]. Ambiguities in the definition and criteria of death have compelled scholars to re-address the moral permissibility of organ donation in Abrahamic religions [2,9-12].

End-of-life organ donation remains controversial in Islam [2,13]. This controversy emanates from: (1) scientifically ambiguous medical criteria of death determination [4-8,14,15]; (2) invasive perimortem procedures for preserving transplantable organs [13,16,17]; and (3) incomplete disclosure of information to consenting donors and families [4,8,18,19]. We have summarized elsewhere the scientific evidence challenging the validity of the 2 alternative criteria of death and recommended that the medical criteria of death should be restored to reflect the singularity of death as a biological phenomenon [20]. We also outlined examples of utilitarian practices in end-of-life organ donation that conflict with religious values and traditional rituals in dying Muslim patients [13,20]. Here, we limit the term “utilitarianism” to mean “persons are used in the same way as things are used” [21].

Sharif et al. described factors influencing the willingness toward organ donation in an international quantitative survey of Muslims (n = 675) “residing in Western countries (United Kingdom, Europe, North America, and Oceanic geography)”: “[t]he main constraints cited by Western Muslims were interpretation of religious scriptures [the Quran and the Hadith] (76.5%) and advice from local mosque (70.2%)” [22].

Gauher et al. [23] also commented on the significance of religious and cultural barriers to organ donation among UK Muslims. Transplant advocates [22-29] have proposed corrective interventions: (1) reinterpreting religious scriptures, (2) reeducating faith leaders, and (3) utilizing media campaigns to overcome religious barriers to organ donation in Muslim communities. Several commentators in Western countries have indeed attempted to reinterpret the Islamic moral code [30-33]. In this article, we focus on: (1) the phenomena of life and death within religious scriptures, (2) the utilitarian interpretations of the moral code in end-of-life organ donation, (3) the societal consequences of such challengeable interpretations, and (4) the targeting of faith leaders with reeducation campaigns promoting these interpretations in Muslim communities.

### The natural phenomena of life and death within religious scriptures

The Quran and the Sunnah are the 2 primary sources of religious teachings and knowledge in Islam [13,34]. The Quran has described the “natural” phenomena of both life and death 14 centuries ago. However, because of a limited capacity to fully comprehend the Quranic verses on these phenomena, scholars continue to be challenged in understanding these descriptions. For example, the Quran describes human development through the early stages of life [35]. Medical embryology has clarified different embryonal and fetal stages of development. Similarly, the Quran also describes the dying process and transition from life to death. The Quran differentiates between the dying process and death:

“Then why do you not (intervene) when (the soul of a dying person) reaches the throat? (83) And you at the moment are looking on, (84) But We (i.e. Our angels who take the soul) are nearer to him than you, but you see not (85)” (56: 83–85) [36].

**Figure 1:**
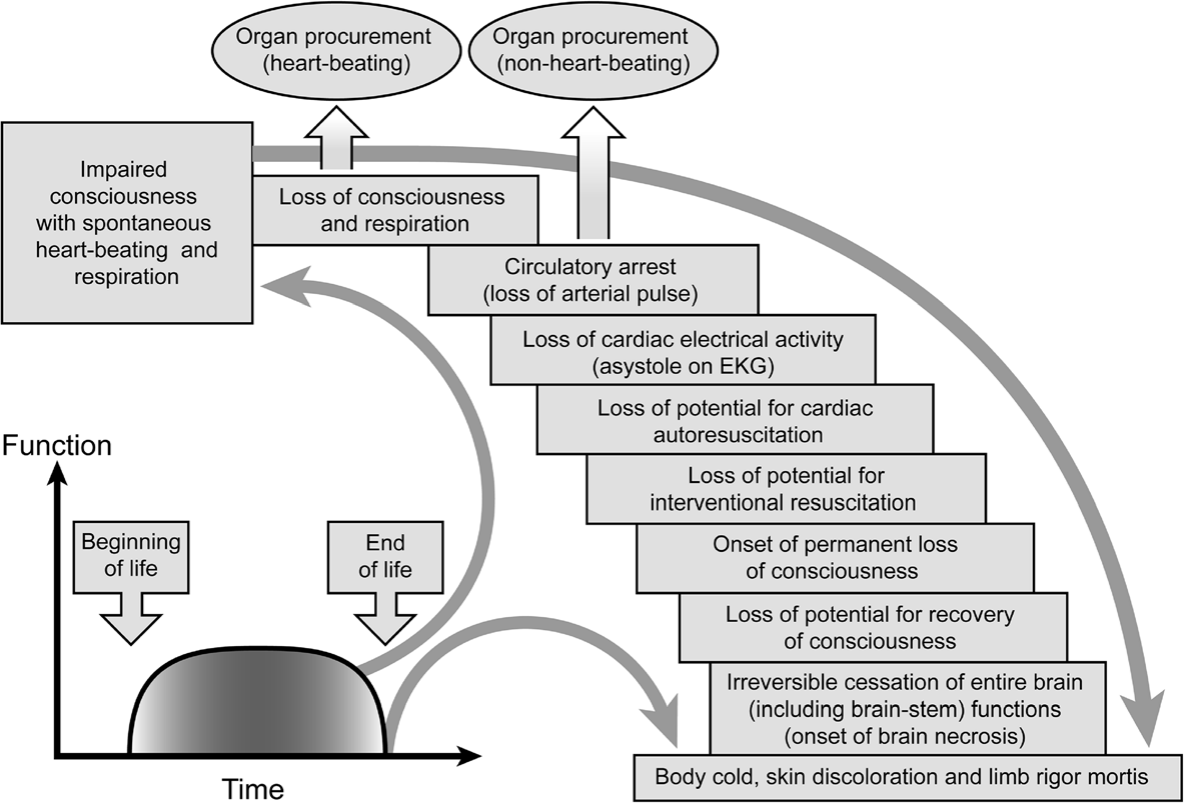
**Human death is a singular phenomenon.** “Human death is a singular phenomenon. The dying process occurs in stages over time. There is a gradual loss of capacity for somatic integration of the whole body because of an irreversible cessation of all vital and biological functions including circulation, respiration (controlled by the brainstem), and consciousness. The irreversibility of cessation of circulatory and respiratory functions is interlinked to the onset of whole brain necrosis. The loss of capacity for consciousness is irreversible when the necrosis of the whole brain, including the brainstem, is complete” [37]. Disintegration begins after completion of the dying process. There is no accurate clinical test to ascertain the absence of self and/or environmental awareness in unresponsive patients following severe brain injuries. Arbitrary neurological and circulatory criteria redefining human death enable heart-beating and non–heart-beating procurement of transplantable organs, respectively. Scientifically flawed criteria of death can harm donors because procurement procedures are performed without general anaesthesia. Figure reproduced from source [37], under the terms of the Creative Commons Attribution License (http://creativecommons.org/licenses/by/3.0/).

Advances in resuscitation science appears to corroborate the Quranic characterization of the dying process. Different stages in the dying process can be discerned before death (Figure 1) [37]. The complete loss of vital organs’ capacity to recover their respective functions completes the dying process and death follows as a final, singular and irreversible event [13]. The body begins disintegration at death. The Quran describes the disintegration process: “Who will give life to these bones after they are rotten and have become dust?” (36: 78) [36].

To facilitate organ donation and transplantation, contemporary transplantation practices use 2 types of death [1]. Death is now determined by either the brain criterion (ie, brain or neurological death) in heart-beating organ procurement or the circulatory criterion (ie, cardiorespiratory death) in non–heart-beating organ procurement (Figure 1). Scientific evidence has challenged both criteria of death [1,4-8,14]. Either criterion is inconsistent with biological death because: (1) donors determined dead by the neurological criterion can retain normal coordination of bodily physiological functions and/or critical brain functions that are characteristic of living human beings, and (2) donors determined dead by the circulatory criterion can retain viable central brain pathways and neurological responsiveness [1,4,6,7,15,38]. Currently, there is no accurate clinical test that can ascertain the absence of awareness following severe brain injuries. Advances in neurosciences suggest that the capacity for consciousness and self-awareness can be retained despite extensive injury to the human brain [39-42]. Donors retaining viable central neural pathways also may experience nociception during surgical procedures [1,7,43,44]. Living human beings suffer when surgical procedures are performed without general anesthesia. The Quran (as do many scientists and scholars) affirms that death is a singular event, and therefore applying current medical criteria of death can inflict harm onto organ donors [13,20].

### Utilitarian interpretation of the moral code of Islam in end-of-life organ donation

Social contexts may be considered in the interpretation of the Islamic moral code about human acts that are not mentioned in the Quran or Sunnah. To ratify a moral and legal opinion or fatwa about such acts, qualified scholars apply secondary sources or principles in a process called *ijtihad* (Table 1) [34]. There are 2 preconditions for the validation of an opinion or fatwa: (1) it must not clash with the Quran and the Sunnah, and (2) it must not harm the person’s religion, life, mind, property or progeny (ie, the objectives or *maqasid* of the Islamic law) [34].

**Table 1:** Primary and secondary sources of the Islamic legal and moral code

• Primary sources	○ The Quran: revelation from God to man (first source of Islamic law)
	○ The Sunnah: the tradition of the Prophet Muhammad: what he said, what he did, what he saw and approved during his lifetime (second source of Islamic Law)
• Secondary sources (reinterpretation of the primary sources)	○ *Ijma*: consensus agreement about the moral and/or legal assessment of an act or practice (third source of Islamic law)
	○ *Qiyas*: juristic reasoning by analogy (fourth source of Islamic law)
	○ *Istishab*: the principle of presumption in the laws of evidence that a given state of affairs known to be true in the past still continues to exist until the contrary is proved
	○ *Maslaha*: the principle of reasoning based on public welfare and interest
	○ *Istihsan*: the principle of reasoning based on preference, ie, “seeking to do good”
	○ *Urf*: the principle of reasoning based on customary practice

Table is developed from the source [34]. The primary sources of Islamic law and moral code are the Quran and Sunnah. Secondary sources can be applied to issue legal and moral opinions about acts or practices that are not mentioned explicitly in the primary sources. This process is called *ijtihad*. Sunni and Shiite sects agree on the Quran, Sunnah and Ijma as sources of Islamic law in that order. The Shiite sect considers *Aql* (human intellect) as the fourth source of Islamic law instead of *Qiyas*. Legal and moral opinions or fatwas must uphold the primary objectives or *maqasid* of Islamic law ie, the protection of a person’s religion, life, mind, property and progeny. The application of secondary sources (eg, *maslaha, istihsan*) in end-of-life organ donation is preconditioned that death is determined with an absolute certainty or *yaqin* in accordance with the Quran and Sunnah.

The moral code is intended to protect the inviolability and the dignity of human life regardless of time and place. Interpretation of religious scriptures to justify a medical practice perceived to be socially desirable, without the prerequisite observance of the *objectives* of Islamic law, will transgress the moral code. We contend that redefining death in end-of-life organ donation is an example of such misaligned interpretation. For 4 decades, the scientific controversy has continued on death determination in organ donation [1,4]. A social construct of death may well serve utilitarian objectives in society, that is, donors are categorized as dead so that procured organs are transplantable into other living humans. However, such a utilitarian social construct of death can harm donors and pose moral challenges to the transplantation practice [7,38].

The Council of Islamic Jurisprudence accepted brain death as biological and legal death in 1986 [45]. Since then, the majority of Islamic institutions and councils in Western countries have issued legal opinions and fatwas permitting end-of-life organ donation [46]. However, these opinions or fatwas are revocable because: “fatwas are generally acknowledged as fallible opinions because of the possibility of human misunderstanding, misinterpretation or lack of knowledge about the phenomenon which fatwas are addressing” [46]. Indeed, recent advances in the clinicopathological characterization of brain death have mandated a critical reappraisal of past fatwas [47]. Various Muslim scholars have rejected past fatwas on brain death because of theological and medical reasons. Sachedina [48] has commented that brain death is incompatible with the Quranic description of life and death. Sarhill et al. [49] have rejected the criterion of brain death because it is imprecise and contradicts the Quran and Sunnah. Bedir and Aksoy [50], after analyzing scholarly Islamic sources, concluded that brain death is not complete death.

Padela and colleagues [51] reaffirmed the clinical ambiguity in brain death determination. In Islam, the criterion of death must be unequivocal and grounded in robust evidence so as to uphold the inviolability of human life. In an address to the Organ Transplantation Congress in Abu-Dhabi on February 1998, the Muslim scholar Al-Qaradawi reemphasized that “the legal Islamic opinion which is in favour of organ donation and organ transplantation, *as long as we are sure that all the moral and religious conditions have been met*” [emphasis added] [52]. The Quran describes death as *yaqin* (Arabic word for absolute certainty), ie, a singular event determined with an absolute certainty at a specific time: “And worship your Lord until there comes unto you the certainty (i.e. death)” (15:99) [36]. If the medical criterion cannot validate death determination with an *absolute certainty,* then end-of-life organ donation transgresses the moral code. *Al-zann* (ie, doubt, uncertainty, conjecture, or suspicion) is the opposite of *yaqin* and is legally and morally prohibited in death determination. The Quran warns against *al-zann* because it can lead to deviation from the truth: “Certainly, conjecture (al-zann) can be of no avail against the truth” (10:36) [36]. Consequently, acts that are based on *al-zann* can have negative consequences: “Avoid much suspicion (al-zann), indeed some suspicions (al-zann) are sins” (49:12) [36]. Transplant advocates have accepted *al-zann* instead of *yaqin* in death determination [31-33]. They argue that *al-zann al-ghalib* (ie, the dominant probability) is sufficient to justify contemporary practice of organ procurement in brain death. We think that *al-zann al-ghalib* in death determination is a major departure from the moral code because it is grounded in the faulty assumption that early stages in the dying process (Figure 1) are synonymous with death. Medically, the *prognosis* of death is mistaken for the *diagnosis* of death [53,54]. Indeed, Western advocates admit that brain death is a social construct based on equating human death with the permanent loss of personhood rather than of biological life [32,33]. Acceptance of this construct of death conflicts with the Quranic characterization of death and emphasis on the sanctity of life. Since *yaqin* (certainty) is a precondition in death determination, leading Muslim scholars have rejected the neurological criterion of death [55].

Advocates have also argued that the principles of *maslaha* and *istihsan* can justify end-of-life organ donation [30,31,45,52]. However, if death cannot be determined with absolute certainty then these principles are not applicable (Table 1). *Maslaha* and *istihsan* can invoke intrinsically subjective and challengeable opinions. In *maslaha*, saving the lives of persons with end-stage organ disease is considered important for societal welfare. However, *maslaha* cannot justify ending a human life (donors) prematurely to procure transplantable organs since this act transgresses the moral code [20]. In *istihsan,* (Arabic meaning “seeking to do good”), donating an organ is giving another person the “gift of life” and is considered a charitable act. The *istihsan* is based on the good intention and goodness of the act of giving the “gift of life” to another human being. This is also flawed. First, frequently there are alternative medical treatment options available, although less preferred in society, for saving the lives of those with end-organ disease [20]. Second, transplanted organs are not a permanent cure as manifested by the immune system’s ultimate rejection in surviving recipients. Surviving recipients are burdened with serious medical complications that can develop because of anti-rejection (immunosuppression) medications which can be life-threatening [56,57]. Third, it can be argued that the gifting of an organ is counterintuitive to the Islamic belief in divine creation and personal entrustment of the body.

Other principles such as *istishab* (presumption of continuity) also prohibit organ donation in brain death. Badawi [58] describes this principle “…if it is uncertain if a patient is dead (however evolving definition of death is accepted), then continuity of life should be presumed until death otherwise is confirmed”. It follows that procuring organs from donors declared dead with a medically ambiguous criterion is the same as procuring organs from a living human being. Based on the preponderance of evidence that brain-dead persons retain most of the living characteristics of human beings, including some brain functions, the principle of *al-zann al-ghalib* equally prohibits equating brain death with death. The moral code mandates *yaqin* in death determination.

In a joint physician-jurist seminar on brain death and organ donation held in Riyadh, Saudi Arabia on 16 April 2012, Kasule [59] reiterated several Islamic legal principles that may be violated. First, the principle of intention is violated because “organ harvesting, ICU [intensive care unit] costs and research have been a driving force behind development of brain death criteria” [59]. Second, the pressure to declare brain death can be “causing potential harm to a donor to benefit a recipient which would violate the principle that prevention of harm has precedence over getting a benefit” [59]. Third, “the principle of certainty: recognition of death [must] be based on clear evidence…brain death criteria do not reach the level of absolute certainty … [in Islamic] Law doubt does not void a certainty: in this case life is a certainty and brain death is a doubt” [59]. Fourth, “the principle of custom [*Urf*]: consensus on criteria of death … [must] be by a preponderant majority of the professionals and not by a minority… [and] also must have stood the test of time… [brain death] criteria have been changing with development of knowledge and technology and have not reached the level of universal consensus having variation by country and by institution” [59]. Fifth, the construct of brain death transgresses the *maqasid* of Islamic Law ie, “protection of life” and that “*…* death should not be declared in a living person without evidence-based certainty…” because “[m]istaken diagnosis has very severe consequence” [59].

### Societal consequences of utilitarian interpretation of the moral code of Islam

Sharif et al. [22] invoked the principle that necessity overrides prohibition to ameliorate the negative implications from ambiguous death determination:

“*Although violation of the human body, whether alive or dead, is forbidden in Islam a greater emphasis is placed on altruism and humanitarian need.* “If anyone saved a life, it would be as if he saved the life of the whole people” [36] is a shared principle among the Abrahamic religions *and provides theologic justification for enacting the Islamic juristic principle of al-darurat tubih almahzurat or “necessity overrides prohibition”* [Emphasis added]*.*

The implicit argument is that societal needs for organ transplantation should take priority over the prohibition of procuring organs from donors who may not be dead by the biological standard. Khalid and Khalil [60] have commented “[a] dying person may not need his viable organs as much as persons on waiting lists, where an organ transplant could make a difference.” The aforementioned comments can be construed as to mean that expediting the demise of a dying person to procure transplantable organs is morally acceptable. This argument may be acceptable to some people under the conditions that: (1) an open public debate has taken place; and (2) a broad agreement has been established on arbitrarily defining death to facilitate organ donation. Neither condition has been met [4,7,38]. Most in society disagree a priori on the permissibility of procuring organs before biological death. The utilitarian objective in redefining death for organ donation and transplantation is grounded in the notion that “the end justifies the means”. However, Islam and other Abrahamic faiths [2,3,9-11] forbid terminating life for donating transplantable organs with no exceptions made for “altruism and humanitarian need” as suggested by Sharif et al. [22]. The Quran condemns the intentional termination of human life unjustly: “And whoever commits that through aggression and injustice, We shall cast him into the Fire” (4: 30) [36]. Sharif et al. advocated reinterpreting religious scriptures in favor of donating organs at the end of life [22]. They advanced the utilitarian reinterpretation of religious scriptures by citing the Quranic verse that saving one life is as saving the whole of mankind and, therefore, organ donation and transplantation is permissible [22,25]. However, Sharif et al. cited just a portion of the Quranic verse: “if anyone killed a person —not in retaliation of murder, or (and) to spread mischief in the land — it would be as if he killed all mankind, and if anyone saved a life, it would be as if he saved the life of all mankind” (5:32) [36]. Although saving one life is as saving the whole of mankind, the complete verse ranks condemnation of terminating a human life above commendation of saving a life [13]. The ranking of “killing a person” above “saving a life” reaffirms that preventing evil supersedes promoting good [13].

Sharif et al. proposed interventions that are focused on “global Muslim populations”, “demographic groups”, and “influential parties” encouraging positive attitudes toward donation in Muslim communities. In defense of their effort to increase donation rates, they argued that their approach could contribute to societal welfare by preventing “…Western Muslims developing resentment and temptation into organ trafficking” and “Muslims becoming a growing burden on dialysis programs” [22]. The authors conflated the secular moral theories of consequentialism, utilitarianism, and autonomy with religion-based morality. This utilitarian reasoning and concern with self-serving interests may be discernable in transplantation practice. For instance, Sharif circumvented the controversy about the definition of death by reasserting the authority of the medical profession in settling this matter and ignoring (scientific and/or theological) concerns:

“[t]he major issue that stems from such debate is who should be deemed the ultimate authority to determine the eventuality of death—physicians or theologians? It is clear from this discussion that opposing views on the subject of brain death criterion can be broadly but not exclusively categorized into physicians versus theologians (supporters of a physical vs. philosophical definition of death, respectively). In Islam, passing of the body may be different from passing of the soul, but we can only rely on physical rather than metaphysical examination to determine the moment of death. I would argue physicians are the true determinants of cessation of (physical) life because the metaphysical is beyond any human assessment” [25].

Sharif disregarded contemporary scientific objections to the medical criteria of death. He depicted the Quranic characterization of death as a singular phenomenon as merely philosophical (metaphysical) and, therefore, physicians should be the decisive authority in defining death criteria. The assertion that physicians (more precisely, the transplantation community) should be customizing the death criteria, for the goals of the organ transplantation practice, highlights the creep of moral reasoning toward the duty-to-die and utilitarian medical homicide [61,62]. Engelhardt [63] has described the rise of secular moral reasoning in medicine and concluded that it “results from the death of God and the abandonment of a God's eye perspective” in “posttraditional Western societies”. Engelhardt [63] pointed out that controversial end-of-life medical practices have thrived through “secularizing” morality and dismantling traditional moral boundaries of Abrahamic faiths. Indeed, the new field of “Islamic bioethics” appears to be emerging with a focus on reinterpretation of religious text to accommodate utilitarian-based objectives in medicine [64]. History has shown the disastrous consequences of dismantling traditional moral boundaries under the premise that the medical profession knows what is best [65,66]. Utilitarian medical objectives culminated in extermination of “vulnerable populations” under the premise of compassionate care, relief of human suffering, and societal welfare [66]. The “moral vulnerabilities” and utilitarian pressures that previously justified prematurely ending “life unworthy of life”, are still thriving in “contemporary medical culture” [67]. Hamdy [68] has cautioned against the slippery slope in utilitarian transplantation practice by describing an example from Germany: “…many books were published in Germany voicing fears and criticism of organ harvesting from brain-dead patients” because of “haunted memories of state violence and its link to biomedical practice.” Therefore, it is surprising that Islamic councils continue to rely on the premise that “the medical profession is the proper authority to define the signs of death” [46] without critically evaluating the scientific validity or the utilitarian objectives underlying the death criteria.

We have cautioned of the sociocultural consequences of reinterpreting the moral code to conform to the utilitarian ideology of increasing end-of-life organ donation [13,20]. Critics of this utilitarian ideology are generally disdained:

“when people in other societies voice antipathy toward medical procedures like organ procurement, they explain their own stance in terms of “culture”, such that similar feelings of antipathy from within the US dominant culture are rendered imperceptible” [68].

The dominance of utilitarian ideology in Western countries is evident when Randhawa [69], a member of the UK Organ Donation Taskforce, rejects the relevance of religious scriptures because its moral code is dictated by values that are ancient and irrelevant in modern day practice of organ transplantation:

“We need to acknowledge that most religious scriptures *were written* hundreds, if not thousands of years ago, before any consideration of organ transplantation. Consequently, any religious position on organ donation is subject to a religious scholar’s interpretation of the scriptures and *the values espoused by the faith*.” [Emphasis added].

Theologians would disagree with Randhawa because of the theological view that religious values of the Abrahamic faiths are held to originate from one divine source and apply regardless of time. Along the same line of reasoning, Moosa has counter-argued theological dissent: “theologians and pseudo-theologians use science… in order to prove the validity and the wisdom of their scriptures only to put on display the ‘truth’ of their respective faiths.…these pseudo-theologies are embarrassing for both serious scientists and theologians” [32]. However, it can be argued that the so-called “serious scientists and theologians”, who are rejecting the truth and wisdom of the religious scriptures, are imposing their societal ideological preferences and values over those of others. Padela and Zaganjor have blamed negative attitudes towards organ donation on “negative religious coping” as well as an “insecure relationship with God and an ominous view of the world” [70]. Advocates appear to overlook that the Quran is held to be the ultimate reference in Islam to settle disputes on the moral boundaries of human behavior: “He sent the Scripture in truth to judge between people in matters wherein they differed” (2:213) [36].

In spite of valid scientific and theological objections, many Sunni and Shiite scholars continue to espouse the permissibility of end-of-life organ donation [71,72]. One implication of reinterpreting religious text for the benefit of a utilitarian transplantation practice is that religion’s primary value, ie, the sanctity of life and human dignity is being violated. Incorrect reinterpretation of the moral code by special interest groups transgresses the religious rights of Muslim communities and constitutes intellectual violence against religious scholarship. The Quran cautions against deviant interpretation of the moral code to appease special interests: “And if the truth had been in accordance with their desires, verily, the heavens and the earth, and whosoever is therein would have been corrupted!” (23:71) [36].

### Media campaigns and reeducation of Muslim faith leaders

Sharif et al. [22] conveyed conflicting messages about Islam. They stated “…poor donation consent among Western Muslims centered primarily around theologic pressures, from interpretation of religious scriptures or advice from Imam or Mosque” to then attribute this unfavorable interpretation or advice to “the lack of education and/or awareness” [22]. Sharif et al. suggested 2 related strategies: reeducating religious leaders and utilizing media campaigns. For example, Sharif et al. disapproved of 2 religious beliefs prevalent in Muslim communities: (1) the forbiddance of physically violating the living or deceased human body, and (2) the “fatalist attitudes of predetermination” prohibiting “human interference to alter a preordained course of medical events” [22]. They connoted these 2 religious beliefs that are barriers to willingness to organ donation *as lack of education*. Others would argue these religious beliefs are founded on: (1) the sanctity of God’s creation of life and human body, and (2) the divine predetermination, Will and Decree (*Al-Qadaa wa Al-Qadar*). The Quran is the source of these beliefs: “Verily, We have created all things with Qadar (Divine Preordainments of all things before their creation, as written in the Book of Decrees)” (54:49) [36].

We have addressed elsewhere how mass media can be an effective tool of communication to remove religious barriers toward organ donation [18,73]. For example, selective disclosure of information in media and educational campaigns promoting organ donation in Muslim communities is not novel. Scholarly and scientific debates that are critical about organ donation are generally excluded from educational and media campaigns. Yilmaz applied similar strategies to improve willingness to organ donation among Turkish Muslims [24]. He found that religious interpretation of death was the main reason for refusing organ donation [24]. Yilmaz applied 2 consecutive interventions in a pilot study of 132 Muslim men so that “…wrong beliefs about organ donation disappeared”: (1) a one-hour teaching session on favorable reinterpretation of religious scripture about death and organ donation; and then (2) a continuous exposure to favorable public messages about organ donation in multimedia campaigns (eg, educational brochures and posters) for a period of 2 months [24]. The refusal rate of organ donation decreased from 54% at the beginning of the study to 17% after the completion of the study [24]. Yilmaz proved that controlling the information in media campaigns could influence willingness to organ donation. Yilmaz selectively disclosed information on “opinion of Supreme Board of Religious Affairs” that was favorable on brain death and organ donation in his study. He did not inform the study subjects that Turkish Muslim scholars [50] have rejected brain death as the Islamic definition of death. In another Turkish survey in the province of Kayseri, Guden et al. [29] considered overcoming the unfavorable attitudes among religious officials (Imams, muezzins, preachers, and Quran educators) toward organ donation by mischaracterizing scientific and medical concerns as “social reflex of skeptics”:

“As known, officials of religion preach people and answer questions from the Islamic point of view and provide interpretations. People pay attention to these interpretations from the officials according to their religious vulnerability and behave accordingly. It is of utmost importance that officials of religion explain that their *religious reasons opposing organ donation are not valid, especially to those who are skeptical about organ donation and show an opposing attitude as a social reflex but ground it on religious beliefs*” [Emphasis added].

Turkyilmaz et al. [28] proposed a similar strategy of targeting Muslim religious officials in the Eastern Black Sea region of Turkey with “appropriate education” to improve willingness to organ donation. In a subsequent survey of religious officials, Tarhan et al. reported 92% had favorable views to organ donation [74]. Nondisclosure of the medical, legal, and religious controversies about the death criteria and organ donation was a common theme in public surveys [27-29,71,72,74,75]. Surveyors did not discern willingness to organ donation if the death criteria were inconsistent with the Islamic moral code. Dissemination of incomplete information violates the ethical principles of transparency and truthfulness in medicine and denies individuals the right to informed decision making. It is not surprising that almost 24% of US physicians object to donation because of concerns about the quality of end-of-life care and the invasiveness of perimortem procedures associated with organ procurement [76]. We recommend that medical information about how death is determined and the surgical procedures that are performed for organ procurement should be communicated clearly and explicitly to the general public in media campaigns and opinion surveys. Attempting to influence behavior and attitudes through disclosing incomplete information or communicating incorrect interpretations contradicts the Islamic moral virtues of truthfulness and honesty.

## Conclusions

Proposals by organ transplantation advocates to promote organ donation in Muslim communities disregard the appropriate application of Islamic jurisprudence and the growing scientific and theological controversies in Western societies about death determination. Utilitarian reinterpretation of the religious scriptures for the purpose of embracing a controversial end-of-life practice, perceived to be socially desirable, has deleterious practical consequences: (1) donors can suffer harm; and (2) utilitarian secular moral reasoning resets the Islamic moral code that uphold the sanctity and dignity of human life.

## Competing interests

The authors declare that they have no competing interests.

## Authors’ contributions

MYR and JLV attest that they have made substantial contributions in drafting the manuscript and revising it critically for important intellectual content, that they have given final approval of the version to be published, and that they have participated sufficiently in the work to take public responsibility for appropriate portions of the content. Both MYR and JLV have read and approved the final manuscript.

## Authors’ information

Dr. Mohamed Rady is Professor of Medicine at the Mayo Clinic College of Medicine and a clinical consultant in the Department of Critical Care Medicine at Mayo Clinic Hospital in Phoenix, Arizona. Dr. Rady served on the Ethics Committee of the American College of Critical Care Medicine. Dr. Joseph Verheijde is Associate Professor of Biomedical Ethics at the Mayo Clinic College of Medicine and Assistant Professor of Physical Therapy in the Department of Physial Medicine and Rehabilitation at Mayo Clinic in Arizona.
